# New Druggable Targets for Rheumatoid Arthritis Based on Insights From Synovial Biology

**DOI:** 10.3389/fimmu.2022.834247

**Published:** 2022-02-21

**Authors:** Gurvisha Sandhu, B. K. Thelma

**Affiliations:** Department of Genetics, University of Delhi, New Delhi, India

**Keywords:** rheumatoid synovium, synovial fibroblast, macrophage, OMICS, signaling, druggable targets

## Abstract

Rheumatoid arthritis (RA) is a multifactorial autoimmune disease characterized by chronic inflammation and destruction of multiple small joints which may lead to systemic complications. Altered immunity *via* pathogenic autoantibodies pre-date clinical symptom development by several years. Incompletely understood range of mechanisms trigger joint-homing, leading to clinically evident articular disease. Advances in therapeutic approaches and understanding pathogenesis have improved prognosis and likely remission. However, partial/non-response to conventional and biologic therapies witnessed in a subset of patients highlights the need for new therapeutics. It is now evident that joint disease chronicity stems from recalcitrant inflammatory synovial environment, majorly maintained by epigenetically and metabolically reprogrammed synoviocytes. Therefore, interference with effector functions of activated cell types seems a rational strategy to reinstate synovial homeostasis and complement existing anti-inflammatory interventions to mitigate chronic RA. Presenting this newer aspect of fibroblast-like synoviocytes and myeloid cells underlying the altered synovial biology in RA and its potential for identification of new druggable targets is attempted in this review. Major leads from i) molecular insights of pathogenic cell types from hypothesis free OMICS approaches; ii) hierarchy of their dysregulated signaling pathways; and iii) knowledge of druggability of molecular nodes in these pathways are highlighted. Development of such synovial biology-directed therapeutics hold promise for an enriched drug repertoire for RA.

## 1 Introduction

Rheumatoid arthritis (RA) is a common chronic inflammatory disease of autoimmune nature, affecting ~1% of the population worldwide, with females being more often affected than males (1). It is a symmetric polyarticular arthritis, typically localized distally in small joints of the hands and feet and if left untreated, leading to progressive articular damage, disability and comorbidities over time. Although systemic autoreactivity and immune dysregulation, both innate and adaptive, are predominant phenomena implicated in RA onset (2), precise mechanisms leading to transformation into a joint-specific disease are still emerging based on immunologic (3) and tissue-specific evidences (4, 5). Current treatment strategies are mostly limited to anti-inflammatory and immune-targeted disease-modifying anti-rheumatic drugs (DMARDs) such as methotrexate and/or biologics. However, both synthetic and biologic drug categories exhibit drawbacks, namely limited efficacy (only 40-50% show lowering of disease activity or remission), contraindications and adverse effects including risk of opportunistic infections (6). Therefore, efforts to develop new therapeutics based on disease biology which may not only address the heterogeneity in drug response but also multiple synovial pathologies in RA are imminent. Application of multi-omics approaches in RA in recent years has elucidated imprinted phenotypic heterogeneity in stromal elements especially fibroblast-like synoviocytes (FLS) that in turn escalate disease progression and severity (7–9). These findings may be insightful for identification of novel potential drug targets. To this effect, design and development of small molecule inhibitors (SMIs) targeting intracellular dysregulated signaling networks in the synovium are a promising therapeutic class, which may also shift the treatment paradigm in RA. This review aims to summarize the etiology and pathogenesis of RA, followed by current treatment limitations, present OMICS-based molecular signatures in rheumatoid synovium and highlight examples of potential druggable targets thereof; and is restricted to seropositive RA which accounts for two-thirds of all disease burden.

## 2 Background: Rheumatoid Arthritis

Genetic predisposition along with multiple crucial encounters with environmental exposures over a time period support a multi-hit time-lag disease model for this multifaceted disease (10). Low concordance rates for RA in monozygotic (15%) and dizygotic (4%) twins imply a major contribution of environmental factors in disease etiology (11) but these remain unclear. Lifestyle factors including diet and obesity, and exposure to smoking, silica dust, microorganisms etc are reported to be strong drivers of the disease (12). Of these, the rather recent epidemiological data support a role for periodontal bacteria *Porphyromonas gingivalis* in RA etiology wherein statistically significant interactions between elevated levels of anti-citrullinated protein antibodies (ACPAs) against its virulence factor, smoking and *HLA-DRB1* shared epitope alleles were observed in ACPA-positive RA (13). Another study involving intestinal microbiota *Prevotella copri* has provided evidence for involvement of molecular mimicry in RA etiology (14). These emerging findings have begun to notably uncover the environmental attributes of RA etiology.

As for the genetic contributors, the earliest and most robust genetic associations remain to be that with HLA genes (15, 16). Disease associated alleles of HLA locus (like HLA-DRB1*01, HLA-DRB1*04 and others), which share common amino acid sequences in the peptide-binding groove termed shared epitope (SE) strongly implicate peptide (and self-peptide) binding in the pathogenesis, across multiple ancestries (16). Over the last few years, more than 100 risk loci significantly associated with RA have been revealed by multi-ethnic case-control based genome-wide association studies (GWASs) (17). However, functional characterization of the disease-associated genes/loci is paramount for understanding their likely role in pathophysiology of RA. A few of these genes are suggestive of contributing smaller functional effects *via* altered co-stimulatory pathways (*CD28*, *CD40*, *CTLA4*), lymphocyte receptor activation (*PTPN22*), signal transduction pathways regulating immune response (*STAT4*, *TNFAIP3*, *TRAF1*) and cytokine signaling (*IL-6R*, *TNF*, *IL-1RA*) (18). Biologics developed against some of these genes for RA treatment (**Table 1**) lend support to their likely role.

**Table 1:** FDA approved therapies available for RA along with major adverse effects.

DMARDs	Binding Target	Administration Route	Processes Affected	Major Adverse Effects/Risk of Infection	References
**Conventional Synthetic DMARDs**					
Methotrexate, Sulfasalazine, Chloroquine, Hydroxy-chloroquine	Unknown targets	Oral/ subcutaneous/ intra-muscular	Immuno-inflammatory reactions by increased adenosine release and binding to cell surface receptors	Gastrointestinal toxicity; hepatic dysregulations; pneumonitis	(19)
**Biological DMARDs**					
Adalimumab, Certolizumab, Etanercept, Golimumab, Infliximab	Tumor necrosis factor (TNF)	Intravenous infusion/ subcutaneous injection	Stromal cell activation, angiogenesis, cytokine and chemokine expression, MMP production	Flares in patients with MS; high risk of *Herpes zoster*; reactivation of tuberculosis	(20)
Tocilizumab, Sarilumab	IL-6R	Intravenous infusion/ subcutaneous injection	T-cell migration and activation, FLS inflammatory response, osteoclast activation	Gastrointestinal perforations; severe liver failure	(21)
Abatacept	T-cell co-stimulation signal (CD80/CD86)	Intravenous infusion/ subcutaneous injection	Effector T-cell and dendritic cell activation, B -cell infiltration, osteoclastogenesis	Moderate chances of serious infections	(20)
Rituximab	CD20 (cell marker expressed on B-cells)	Intravenous infusion	Circulating B-cells, a proportion of tissue B-cells and plasmablasts, autoantibody titers	Risk of *Herpes zoster*; rare risk of progressive multifocal leuko-encephalopathy	(22)
**Targeted Synthetic DMARDs**					
Tofacitinib	JAK1/3	Oral	Cytokine-dependent feedback loops and downstream effects	Risk of venous thromboembolism; *Herpes zoster* (baricitinib, tofacitinib); Hepatitis B reactivation (baricitinib)	(23)
Baricitinib	JAK1/2	Oral			
Upadacitinib, Filgotinib	JAK1	Oral			

MMP, matrix metalloproteinase enzymes; IL-6R, interleukin-6 receptor; FLS, fibroblast-like synoviocytes; JAK, Janus kinase; MS, multiple sclerosis.

Specific interactions between genotypes, environmental risk factors and autoimmunity suggest a molecular basis for breach of tolerance and initiation of pathogenic immune reactions in extra-articular mucosal sites early on during disease development (12). In addition, epigenetic dysregulation of gene expression also leads to altered immune response and disease (24, 25). Based on twin studies, estimated heritability of RA across populations is 53–65% in ACPA-positive RA patients (26–28). Besides major histocompatibility locus (MHC) class II explaining half of it (29) and GWAS-identified susceptibility loci (n=>100) of modest effect sizes (odds ratio<1.5) contributing an additional 5.5% (17), altered epigenome is emerging as a crucial candidate which may account for the missing heritability.

### 2.1 Pathogenesis

**Figure 1:**
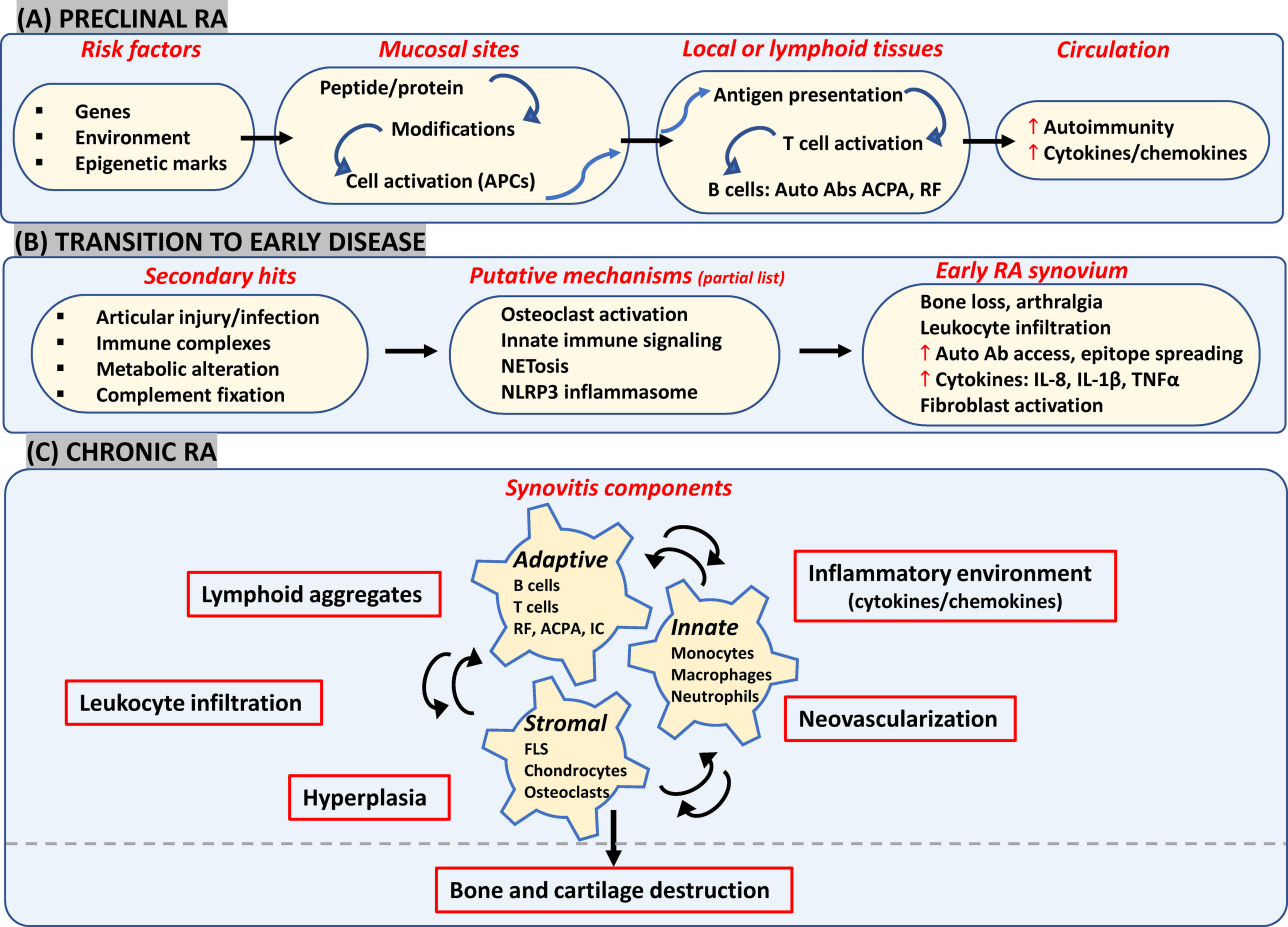
Likely components in emerging view of RA onset and progression. **(A)** Interactions between genetic and environmental factors may trigger post-translational modifications in proteins (such as citrullination and carbamylation) at mucosal surfaces (such as gut, lungs and joints). In a genetically susceptible individual, this can activate innate immunity leading eventually to presentation of modified proteins by APCs like dendritic cells to T-cells in secondary lymphoid tissues. Subsequent systemic autoimmunity is characteristic of preclinical asymptomatic RA. **(B)** Multiple secondary hits may trigger mechanisms leading to synovial inflammation. Local osteoclast activation by ACPAs may cause early arthralgia and leukocyte recruitment. Local tissue insults, immune complex (IC) formation and complement activation may trigger synovial innate cells to increase cytokine production and vascular leakage. Release of NETosis and activation of NLRP3 inflammasome in macrophages could be other triggering mechanisms. **(C)** FLS get activated *via* inflammatory mediators released by lymphocytes, neutrophils and myeloid cells which further enhance inflammatory loops. Resulting synovitis works towards bone and joint damage *via* osteoclasts and FLS in intimal lining along with changes in tissue architecture characterized by ectopic lymphoid structures responsible for epitope spreading and angiogenesis to support cellular heterogeneity and effector functions in hypoxic microenvironment. APCs, antigen presenting cells; ACPA, anti-citrullinated protein antibodies; IC, immune complex; NETosis, neutrophil extracellular traps; NLRP3, nucleotide-binding, oligomerization domain (NOD)-like receptor family, pyrin domain containing 3; FLS, fibroblast-like synoviocytes; RF, rheumatoid factor.

Mechanism(s) responsible for the progressive transformation from autoimmunity in extra-articular mucosal surfaces to joint-specific disease manifestation remain elusive. Taking together the recent experimental evidence suggestive of putative diverse pathways contributing to the clinical phenotype across individuals, the emerging view of RA pathogenesis in its different phases is briefly presented below (**Figure 1**), as a prelude to the next section on therapeutics.

#### 2.1.1 Preclinical Phase

Repeated environmental exposures to cigarette smoke/bacterial products etc which most likely act at mucosal surfaces (lungs, oral cavity, gastrointestinal tract) in genetically predisposed individuals may elicit post-translational modifications of peptides/proteins (12) and/or increase the likelihood of epigenetic alterations (25), with inflammatory consequences. Besides induction of local peptidyl arginine deiminases (PADI2/4) triggering citrullination of histones as well as matrix proteins (fibronectin, collagen, vimentin etc), oral microbiota-derived PADIs may also initiate modification of periodontal tissue proteins (12). This in turn leads to activation of innate immunity and causes local inflammation (30). Antigen presenting cells (such as dendritic cells) present citrullinated proteins to T-cells either locally or at secondary lymphoid organs like lymph nodes, thereby stimulating production of autoantibodies like ACPA and/or rheumatoid factors (RF) (31). Elevated serum levels of different autoantibodies along with greater circulating levels of cytokines and chemokines constitute the asymptomatic preclinical stage, detected up to 10 years before onset of clinical symptoms (32).

#### 2.1.2 Transition to Early Symptomatic RA

One of the processes driving the transition to joint disease tested in *in vivo* experiments suggests a direct articular involvement of ACPAs *via* local osteoclast activation. This may contribute to autocrine enhancement of osteoclast maturation resulting in subtle bone changes and arthralgia, as well as infiltration of inflammatory cells into the synovium, both *via* secreted interleukin 8 (IL-8) (4, 33). Other mechanisms may also trigger inflammatory joint disease such as: i) ACPA-based immune complex formation, complement activation or microvascular insult could respectively engage Fc receptors, complement receptors and Toll-like receptors (TLRs) on synovial myeloid cells which may in turn secrete inflammatory and vasoactive mediators and increase autoantibody access to the joint (2); ii) Peripheral blood and synovial neutrophils may be stimulated by ACPAs/RFs along with inflammatory cytokines causing release of neutrophil extracellular traps (NETs) that may augment epitope spreading along with a positive feedback loop of inflammation (3); and iii) Release of mitochondrial DNA and ROS due to cellular injury may also lead to activation of NLRP3 inflammasome in macrophages and T- cells, which in turn accelerates joint inflammation, *via* IL-1β secretion (5).

#### 2.1.3 Chronic RA

All the above-mentioned events could initiate clinical RA synovitis, characterized by vascular permeability, infiltration of leukocytes in synovial sublining, and hyperplasia in intimal lining as a result of increase in number and activation of resident synoviocytes (10). In brief, there is a continuous interplay between recruited innate immunity (involving monocytes, dendritic cells, neutrophils, mast cells), resident synoviocytes and infiltrated adaptive immunity (involving Th1, Th17, B-cells, ACPA and RF immune complexes) which generates a feed-forward loop in the synovium with further creation of local neoepitopes, activation of cytokine networks and soluble mediators like pro-oxidative mediators, chemotactic molecules, adhesion molecules and proteases. Synovitis may also involve architectural reorganization and neogenesis of ectopic lymphoid structures (ELS), to sustain auto-reactive adaptive immune responses toward locally displayed antigens (34). Neoangiogenesis induced in the inflamed synovium by local hypoxia, cytokines and pro-angiogenic factors (such as VEGF) may also promote cell infiltration (35). As a result, the synovial membrane transforms into an autonomous tissue (pannus) leading to destruction of bone and cartilage (36). FLS, as a part of pannus, transforms into a cancer-like phenotype thereby producing MMPs and mediating cartilage damage. They also migrate to other joints spreading destructive arthritis in a symmetrical manner. Bone destruction is primarily mediated by osteoclasts activated by ‘receptor activator of nuclear factor-κB’ (RANKL) and inflammatory cytokines in the synovial lining (10). If clinically manifested RA remains untreated or becomes treatment refractory, it can lead to extra-articular manifestations such as cardiovascular disease (CVD), interstitial lung disease, and increased risk of malignancy, due to increased immune complexes and acute phase response (37).

## 3 Caveats in RA Management and the Way Forward

Past two decades have witnessed tremendous innovations and significant changes in the management of RA. The first step was symptomatic treatment with non-steroidal anti-inflammatory drugs (NSAIDs) to relieve pain and swelling, and glucocorticoids to provide anti-inflammatory effect, although their adverse effects preclude their long-term use. RA management has since shifted to targeting the underlying immune-inflammatory processes by DMARDs (38). A combination of early diagnosis and start of conventional synthetic DMARD (csDMARD) therapy, inclusion of low-dose glucocorticoids, consideration of methotrexate (MTX) as the anchor drug in RA, development and inclusion of biological DMARDs (bDMARDs) and targeted synthetic DMARDs (tsDMARDs) to slow down or arrest joint destruction have been the major interventions in the past decades (39–41). In addition, the strategic treat-to-target principles and their updation over the years have been advocated in clinical practice for improved outcomes in RA patients of early (< 6 months) or established (≥ 6 months) disease. This approach includes the rationale of defining a target, namely clinical remission or low-disease activity (LDA) in case of long standing disease, initiating aggressive therapy soon after diagnosis, and applying prompt therapeutic adaptations with set timeframes (such as 3 months) to reach the target, along with regular assessment of disease activity (e.g. DAS28 ranging from >5.1 representing high disease activity, >2.6-3.2 for LDA, and ≤2.6 for clinical remission), and patient risk to comorbidities (42, 43). A tabulated account of current therapies (**Table 1**); and the lacunae in disease management are presented below.

### 3.1 Available Therapies and Their Limitations

MTX monotherapy results in adequate reduction in clinical symptoms and joint damage in about 25-40% of patients; which increases to almost 50% on glucocorticoid addition (6). Around 10-30% discontinue therapy within a year due to adverse effects (44). Introduction of bDMARDs, particularly in combination with csDMARDs, have a greater effect on disease activity and radiographic joint damage, and hence improved clinical outcomes (45). These can be biological originators or biosimilars targeting soluble extracellular and cell-membrane-associated proteins with high specificity (41). Besides cost-effectiveness and affordability being major concerns, approximately 40-44% of patients on bDMARDs such as anti-TNF do not achieve a substantial clinical response and only a small proportion achieve disease remission (46). There is also appreciation of tsDMARDs as newer treatment for RA, that modulate cytokine responses by interfering with intracellular signaling enzymes such as Janus kinase inhibitors (JAKi) (47). However, due to ubiquity of JAK-STATs in cell physiology besides the immune system, risk of side-effects needs consideration. Even after multiple DMARD therapy switching, about ~20%–30% of these patients remain treatment refractory (48) and since there are no biomarkers for treatment response to individual therapeutic agents (49), new therapeutics are the only promise. Furthermore, perspectives gained from treatment response suggest that all biologics, inspite of different targets, offer similar efficacy in combination with methotrexate (ACR70 response rates around 35-40%) which decreases with increase in previous drug exposure (6). Immune cell-directed and anti-cytokine therapy may work by ultimately interfering with a common downstream TNF-α or IL-6 pathway (50). On the other hand, the epigenetic profile of synovial tissue suggests that sustained activated phenotypes of resident FLS and inflammatory macrophages contribute majorly to the perpetuation of inflammatory microenvironment in the synovium, thereby leading to chronic RA or risk of relapse on therapy lay off (51, 52). Imprinted phenotype of RA FLS persists even on its removal from the cytokine-rich environment of the joint (53). Therefore, solely targeting TNF-α or IL-6 pathways may not be sufficient to restore the cellular and immunologic balance in affected synovial tissue, especially in chronic disease. Appreciation of this limitation yet again highlights the need for new therapeutics. There is also a profound lack of reproducible clinical/biological markers for informed treatment, prediction of response and toxicity, which has serious implications for effective disease management.

### 3.2 The Way Forward: New Drug Targets

The strategy to address this lacuna in the practice of personalized medicine in RA is twofold - namely biomarkers and new druggable targets. Since synovium is the ultimate target tissue in RA, use of high-throughput hypothesis-free OMICS technologies and integrative analyses of synovial tissue may aid biomarker discovery (54, 55). Furthermore, targeted efforts to identify surrogate biomarkers of synovitis in peripheral blood, may be fruitful (56). Biomarker discovery may enable desired predictive, personalized and cost-effective RA management. However, biomarkers in RA are not the focus of this review. Identification of new drug targets, demands a change in focus to vulnerable protein nodes in dysregulated signaling networks operating in aggressive cell types of rheumatoid synovium. Essentially, the armamentarium of therapeutic interventions should be enriched with molecules designed to specifically target these druggable nodes which would in turn affect multiple pathologies manifested in the synovial joint (including hyperplasia, angiogenesis, osteoclastogenesis, cellular infiltration). We propose that such an approach can help tackle chronic as well as refractory RA which is the primary clinical need of the hour. At the same time, preference should be accorded to therapeutic compliance of designed molecules, with respect to route of administration (oral or parenteral), existing comorbidities and risk of contraindications from long-term use, apart from their favorable pharmacoeconomic impact. SMIs are an apropos bet as they are orally administered and target intracellular signaling to offer wide protection against pro-inflammatory cytokines, in contrast to bDMARDs, which block specific extracellular molecules. Position of SMI in therapeutic hierarchy of RA has not been strong, with only JAKi in the market and a litany of failed attempts such as MAPK p38i (57). However, the scenario is now improving with preclinical studies of molecules designed to be more specific on targeted cells and pathways, thereby positively tweaking the efficacy-safety profile of small molecule therapy (58–62). Such efforts are expected to lead to clearing the perpetuating inflammatory damage in the joint and complement efficacy of bDMARDs in order to reset productive immunity.

## 4 Synovial Biology and RA

Based on the preceding sections, it is evident that analyses of diverse cellular and molecular signatures from RA synovial tissue are paramount to understand resistance to available therapies and to select appropriate targets for drug discovery efforts. Some important aspects of synovium in this direction are therefore briefly described below.

### 4.1 Synovium

**Figure 2:**
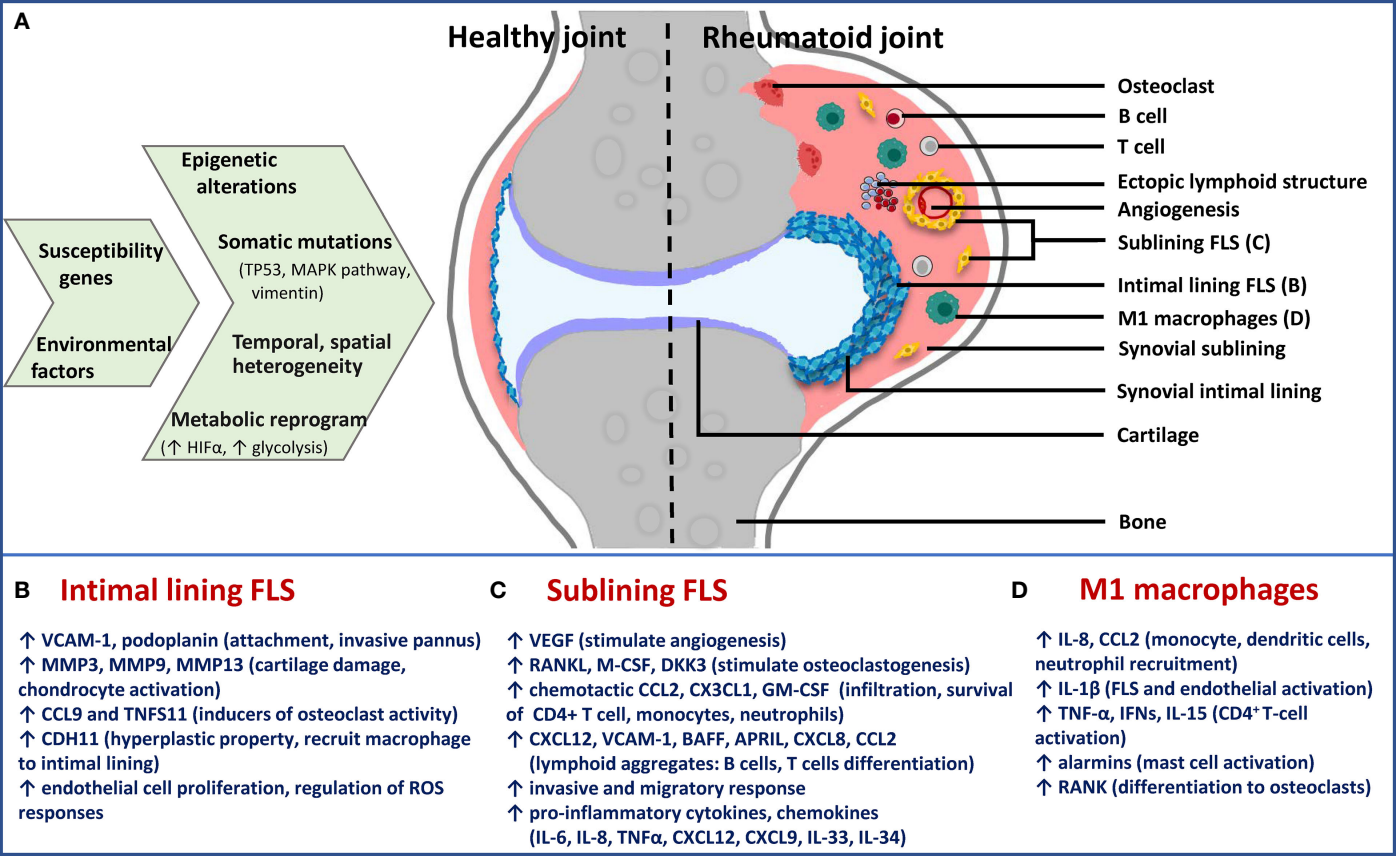
Schematic presentation of cause-effect relationship in RA synovium with specific focus on FLS phenotypes. **(A)** Epigenetic and metabolic reprogramming in resident and infiltrated cell types leads to spatiotemporal and functional heterogeneity in rheumatoid synovium. Difference between healthy and rheumatoid joint is diagrammatically depicted to illustrate changes in synovial architecture like increased angiogenesis and formation of ectopic lymphoid structures, as well as the different cell types. Functionally distinct fibroblast subsets: **(B)** Lining FLS; **(C)** sublining FLS; as well as **(D)** inflammatory (M1) macrophages are the major players in chronic RA. For each of them, effector functions *via* secreted inflammatory and destructive mediators are mentioned below the diagram. Immune-effector fibroblasts and macrophages of sublining are responsible for accelerating inflammatory feed-forward loops, whereas lining fibroblasts mediate bone and cartilage damage. TP53, tumor protein p53; MAPK, mitogen-activated protein kinase; HIFα, hypoxia-inducible factor alpha; FLS, fibroblast-like synoviocytes; M1, inflammatory; VCAM-1, vascular cell adhesion molecule 1; MMP, matrix metalloproteinase; CCL, chemokine (C-C motif) ligand; CDH11, cadherin 11; VEGF, vascular endothelial growth factor; RANKL, receptor activator of nuclear factor kappa-B ligand; M-CSF, macrophage colony-stimulating factor; DKK3, dickkopf WNT signaling pathway inhibitor 3; CXCL, chemokine (C-X-C motif) ligand; GM-CSF, granulocyte-macrophage colony stimulating factor; BAFF, B-cell activating factor; APRIL, a proliferation-inducing ligand; IL, interleukin; TNF-α, tumor necrosis factor alpha.

The synovium is a thin membrane encapsulating the joints and supports its mobility. It consists of two layers, the intimal lining layer composed of FLS and macrophage-like synoviocytes, and the sublining layer, which is the underlying connective tissue composed of fibroblasts, fat cells, macrophages and blood vessels (63). In RA, the synovium becomes expanded and healthy synovial lining transforms into invasive hyperplastic pannus, teeming with aggressive fibroblasts, macrophages and osteoclasts (**Figure 2A**). Inflammatory microenvironment of the sublining layer is characteristic of leukocyte infiltration (B-cells, T- cells, monocytes, neutrophils), activation of resident fibroblasts, differentiation of monocytes into macrophages and dendritic cells and osteoclastogenesis, with all processes facilitated by cell-cell interactions, soluble mediators and supported by neovascularization angiogenesis and sometimes ectopic lymphoid aggregates (36).

### 4.2 Phenotypic Heterogeneity at Cellular Level

Bulk and single cell transcriptional analysis have empowered the deconvolution and dynamic analysis of cellular complexity in inflamed RA synovium at a high resolution (8, 9, 64, 65). RA synovium is marked by spatiotemporal heterogeneity in lymphocytic, myeloid and fibroblast cellular populations and by the re-organization of synovial architecture into specialized niches. A combination of single-cell and bulk RNA sequencing and mass cytometry have identified 18 functionally distinct cell phenotypes in active RA synovium, of which many-fold expansion was observed for *THY1* ^+^ *CD34* ^-^ *HLA-DRA* ^hi^ sublining fibroblast and *IL1B* ^+^ *CD11c* ^+^ *CD14* ^+^ pro-inflammatory monocyte populations (64). Transcriptomes also uncovered pro-inflammatory phenotype of *MerTK^-^CD48^-^* macrophages present in increased proportions in both treatment-naïve and treatment-resistant active RA (65). Although direct therapeutic potential of these dominant cellular populations has not been explored, maximum upregulation of their genes in leukocyte-rich RA synovia suggest that they are a rich source of potential therapeutic targets. These in turn may direct treatment modalities which may be most effective in restoring synovial homeostasis (66, 67). This prospect is clarified further by a brief description of the molecular hallmarks of synovial fibroblasts and myeloid cells, together with the effects they have on their microenvironment in the following section.

### 4.3 Synovial Fibroblasts

Pathogenic potential of synovial fibroblasts is attributed to their anatomically-specialized, functionally non-overlapping, aggressive broad phenotypes that contribute not only to joint and cartilage destruction but also to immune-inflammatory regulation (**Figures 2A–C**). Earlier thought to be mere passive responders to proinflammatory stimuli driven by innate and adaptive immunity, FLS have shown persistent erosion of human cartilage when co-implanted in SCID mice much after removal from the inflammatory environment of RA joint (53), exemplifying their intrinsically acquired activated character. They also display tumour-like features including reduced contact inhibition, resistance to apoptosis, ability to migrate and metastasize *in vivo*. The aggressive behavior of FLS in RA is multifactorial, with major contributors being epigenetic imprinting as well as somatic mutations in key genes, both affecting gene expression and cell phenotype (68). Epigenetic landscape in RA FLS provides evidence that plasticity in methylome signature, histone acetylation and miRNA expression start early in the disease and gets fine-tuned with disease progression. This provides the cell type an activated phenotype in a context-dependent (stimulus, anatomical site) manner, thereby contributing significantly to perpetuation of RA and variability in disease severity (69). Statistically-significant hypomethylation patterns exist in pathways involved with innate immunity (Toll-like receptor signaling) and cell movement (focal adhesion pathway and leukocyte trans-endothelial migration) in late RA FLS (7), making these pathways a prime hub for identification of candidate genes that affect disease progression, besides NF-kappa B (NFĸB) signaling identified from transcriptomics study (64).

### 4.4 Myeloid Cells

Single-cell transcriptome sequencing and immunometabolism studies have revealed distinct synovial tissue macrophage subpopulations varying in their homeostatic, regulatory and inflammatory functions (65). Broadly, they exist on a wide spectrum between M1 (pro-inflammatory) and M2 (anti-inflammatory) phenotypes with a major tilt towards M1 in RA synovium. Under hypoxic environment of inflamed joint, phenomena like Warburg effect, accumulation of glutaminolysis-derived metabolites (succinate, fumarate) and crosstalk between TNF-α and type 1 interferon lead to polarization in type 1 inflammatory macrophages and monocytes towards an activated phenotype. This is mainly mediated by epigenetic rewiring in the enhancer repertoire of signaling pathways involved in immune response, leukocyte migration, PI3K signaling involved in chemotaxis and inflammatory responses to TLR stimulation (52, 70, 71). Moreover, innate cell phenotypes such as M1 macrophages and *IL1B* ^+^ *CD11c* ^+^ *CD14* ^+^ monocytes from RA joints show decreased apoptotic rates compared to those from healthy controls due to microRNA-155 overexpression (72). To sum up, M1 macrophages, resident or those differentiated from infiltrated monocytes, become activated as a result of metabolic and epigenetic rewiring in RA pathogenesis. Their direct and indirect effector functions (**Figures 2A, D**) in the sublining layer drive sustained joint inflammation and damage, along with synovial fibroblasts (66), thereby highlighting a consideration for their druggability. Some cues from contemporary approaches but specifically limiting to these two cell types are discussed in the final section.

## 5 Drug Targets From OMICS Approaches

**Figure 3:**
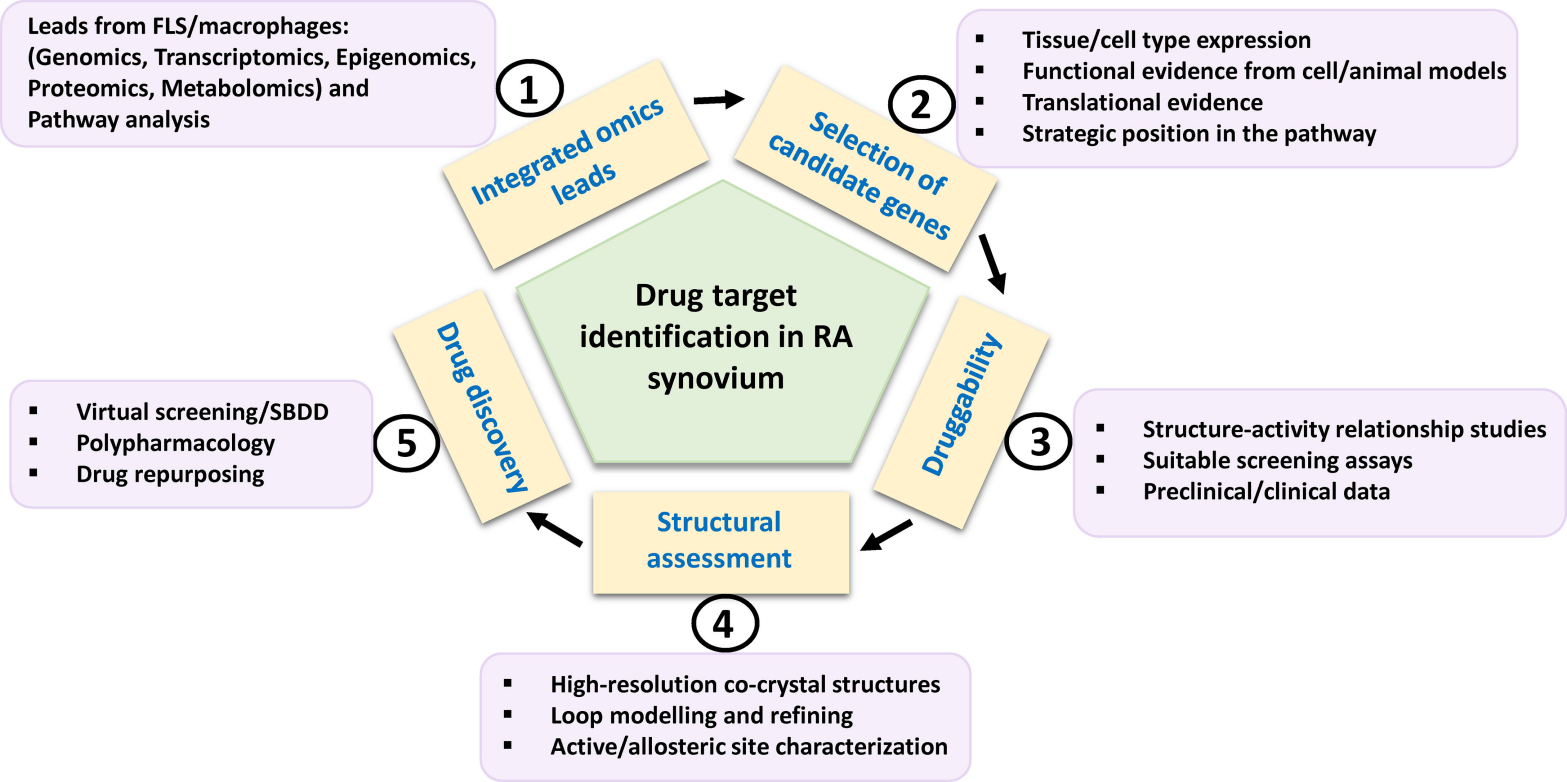
Schema for potential drug target identification from players in RA synovium. Path from omics-derived candidate genes in FLS and inflammatory macrophages to new therapeutics for RA is based on selection of candidate genes on the basis of various factors such as functional evidences. Their amenability to drug discovery may be assessed by preclinical and clinical inhibitor studies. Structural parameters such as availability, refining of functional domain structures and physicochemical properties of binding site residues need to be considered for computational drug discovery involving virtual high throughput screening of chemical library databases or SBDD approach. FLS, fibroblast-like synoviocytes; SBDD, structure-based drug design.

Translational research particularly for new drug development in RA has been accelerated by the assessment of dominant cellular endotypes in target tissue based on newer approaches of functional genomics, epigenomics, transcriptomics, proteomics and metabolomics (7, 64, 73, 74). Specific protein leads from these tools, that are clinically validated and strategically positioned in the dysregulated signaling pathways of the innate and stromal cellular milieu mentioned in the preceding paragraph, their druggability and pharmacological aspects of inhibition are discussed. The criteria, in brief, for prioritizing potential targets (**Figure 3**) from integrated OMICS data sets include cell type expression and localization; implications for multiple underlying mechanisms/pathologies; hierarchy in cell specific networks and cross talks; clinical status of each target and structure-activity relationship (SAR) studies of known inhibitors; availability of qualitative *in vitro* assays; and availability of animal model knockouts. Furthermore, profiling of a druggable target requires consideration and assessment of structural parameters using molecular modelling tools (like VMD, Discovery Studio and AMBER simulation package), an important step which seems to be neglected in new drug target identification. High quality protein 3D structures, appropriate length of the functional domain, length and location of any missing amino acid residues and trans-membrane region, active versus inactive conformations of protein, qualitative characterization of binding pocket(s) for physicochemical parameters, like compactness, surface area, depth, main hydrogen bond and ionic interactions between residues and existing molecules, hydrophobicity, optimal charges, presence of bound water molecules, ions and cofactors are some of the features essential in this time and cost-effective computational approach.

### 5.1 Leads From GWASs and Integrated OMICS

Functional interpretation of GWAS findings for disease pathomechanism is a predicament in GWAS-based drug discovery. To establish a link between risk associated SNPs, susceptibility genes in functionally-relevant cell subsets and involvement in biological pathways underlying RA, multiple studies have attempted aligning GWAS results with data from expression quantitative trait loci (eQTL) analysis, cell-specific and global transcriptomic (scRNA seq) profiling, pathway analysis and cross-trait analysis with other autoimmune inflammatory diseases (75–77). In addition, interrogation of epigenetic underpinnings in patient-derived cell types, facilitated by methylation QTL, epigenome-wide association studies (EWASs) and the more recent methylC-capture sequencing (MCC-Seq) methods may further enhance cell- and context-specific functional interpretation of genetic variants like differentially methylated CpG sites in MHC locus in monocytes (78); IL-6 promoter region in PBMCs (79); and PTPN11 enhancer in RA FLS (80). Strong examples of potential drug targets from these approaches include PADI4 and SRC homology-2 domain-containing protein tyrosine phosphatase-2 (SHP-2) (17).

PADI4, with its expression limited to myeloid leukocytes (monocytes and neutrophils), is localized in cytoplasmic granules and nucleus and is responsible for the posttranslational conversion of arginine residues into citrulline in histone and non-histone proteins (81). SNPs in *PADI4* confer significant susceptibility to RA in Asians and Caucasians, although the association is stronger in Asians (82). Colocalization of *PADI4* eQTL rs230188 with eQTL rs2240335 and epigenetic mark in neutrophils is associated with its increased expression (not observed in monocytes) supporting its functional role in neutrophils (83). Notably, in response to infection and inflammation, neutrophils activate PADI4 to post-translationally citrullinate histones and other autoantigens initiating NETosis, which helps in establishing synovial inflammation (3, 84). PADI4-deficient mice reduce the formation of NETs, autoantibodies, and arthritis, indicating its significant role in RA pathogenesis (85) seems a viable target not only for RA, but also for SLE and cancers. Targeting PADI4 with active site inhibitors may have impact not only on citrullination, but also on transcriptional regulation, apoptosis and innate immunity *via* NETosis. Several preclinical reversible (GSK199, GSK484, streptonigrin) and irreversible (F- and Cl-amidine) inhibitors have been tested *in vitro* and *in vivo* and a few co-crystal conformations (such as PDB 4X8G and 4X8C) are also available. Developing selective compounds, with no activity against other PADI family enzymes is the present focus (61).

**Figure 4:**
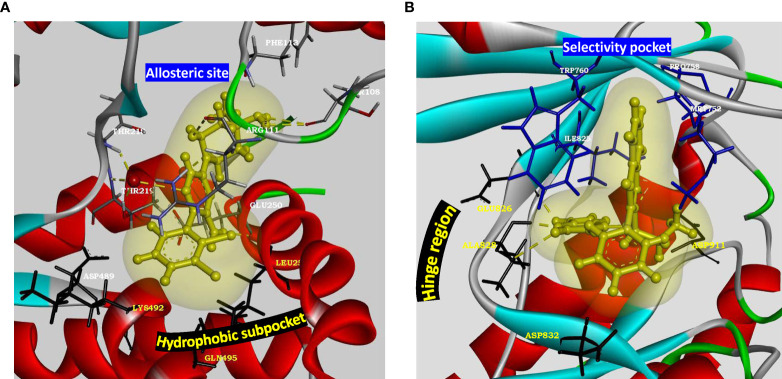
Structural parameters of SHP2 and PI3Kδ for drug design. **(A)** Characterization of allosteric site present in SHP2 (PDB: 5EHR). Key residues of allosteric site (Thr218, Arg111, Phe113 and Thr108) are shown to form water mediated hydrogen bonds with the preclinical inhibitor PCC0208023. Hydrophobic subpocket presents residues Leu254 and Gln495 along with Asp489 and Lys492 for novel pharmacophore design and optimization. Allosteric site residues are rendered as sticks colored by element, hydrophobic pocket residues are rendered as black sticks, oxygen atom of water as red ball and inhibitor as yellow ball and stick model with its soft surface in light yellow and hydrogen bond interactions in yellow dashed lines. **(B)** Characterization of active site of p110 catalytic subunit of PI3Kδ (PDB: 4XE0). Hinge region residues (Glu826, Ala828, Asp832) provide hydrogen bond interactions. Hydrophobic pocket formed by gatekeeper residue Trp760 along with Met752, Pro758 and Ile825 may be targeted for adding selectivity in inhibitor design for delta isoform of PI3K. Kinase domain is rendered as solid ribbon colored according to secondary structure. Hinge region residues are rendered as black sticks, selectivity pocket residues as blue sticks, inhibitor as yellow ball and stick model with its soft surface depicted in light yellow and hydrogen bond interactions as yellow dashed lines.

*PTPN11* is located within a linkage disequilibrium block associated with RA (86) and encodes a cytoplasmic tyrosine phosphatase SHP-2, a known proto-oncogene and cancer target. *SHP2* knockdown in RA FLS cell line inhibited TNF-α induced MAPK signaling (87). Besides *PTPN11* being associated with RA risk (17), a recent study suggested enhanced SHP2 expression in FLS of synovial lining due to abnormal methylation pattern of its enhancer (80). In the same study, SHP2 inhibition in passive K/BxN serum transfer mice model attenuated FLS migration and joint damage thereby demonstrating its role in inflammatory arthritis. Partial pharmacologic inhibition of its phosphatase domain could be a safe and effective strategy for RA treatment. Since SHP2 is directly downstream of growth factor receptors in tyrosine kinase pathway for cell survival and activation, its position in the pathway is strategic and its inhibition may offer a unique advantage in receptor tyrosine kinase drug resistance settings (88). Its co-crystal structures with competitive inhibitors are available, but these compounds (such as NSC-87877, GS-493) show off-target effects due to PDGFRβ binding (89). A recent study has demonstrated inhibition of SHP2/RAS/MAPK signaling pathway and subsequent growth suppression of *KRAS* mutant colorectal cancer cell line, by a non-competitive inhibitor PCC0208023 (90). Based on this observation, it may be proposed that targeting the newly reported SHP2 allosteric site (**Figure 4A**) at the interface between N-terminal SH2, C-terminal SH2 and phosphatase domains is a viable option for developing novel SMI-based therapeutics for inhibiting aggressive phenotype of FLS in rheumatoid synovium lining.

### 5.2 Leads From Epigenomics

Perturbed epigenetic landscape of activated synovial cell types have identified differentially marked genes in innate immune signaling *via* TLR, and pathways for stimulating leukocyte migration and cellular activation (7, 70, 91). Few potential targets from these pathways have been explained such as phosphatidylinositide 3-kinase delta (PI3Kδ), histone deacetylase 3 (HDAC3) in FLS and macrophages and bromodomain and extra-terminal (BET) proteins pertaining to cell activation and mobility (FLS), TLR signaling (FLS and macrophages) and NF-κB pathway mediated inflammation (FLS) as well as cell activation (endothelial cells) respectively.

PI3Kδ, belonging to class I PI3K isoforms, is highly expressed in the synovial intimal lining in RA as compared to osteoarthritis and unlike other isoforms, its expression is markedly induced in FLS by inflammatory cytokines. It is a lipid kinase mediating its biological activity *via* generation of the secondary messenger phosphatidylinositol- (3–5)-triphosphate at the cell membrane, which interacts with several effector proteins. Functional studies have shown that PI3Kδ regulates multiple aspects of pathogenic FLS behavior, like synovial lining hyperplasia *via* AKT activation (92); and platelet-derived growth factor (PDGF)-mediated cell mobility and matrix invasion, through activation of Rac1 (93). Besides FLS, its signaling is also involved with the functioning of multiple immune cells like B-cell activation, cytokine production by T- cells, oxidative burst response in neutrophils and degranulation of mast cells (94), deeming its clinical translation as an exciting and much anticipated proposition. It is also implicated in asthma, chronic obstructive pulmonary disease (COPD), psoriasis and cancer (95). Co-crystal structures of inhibitors with p110δ catalytic subunit of PI3Kδ (such as PDB 4XE0, 6Q74) are available. Extensive SAR and pharmacokinetic profile exploration of different chemical series in comparison to PI3Kδ inhibitor Idelalisib approved for various malignancies, provides starting chemical space for novel drug design with a decent safety profile (94, 96). Linker substitutions targeting the hydrophobic specificity pocket (formed by Trp760, Pro758 and Met752), along with optimization of hinge binder moiety (**Figure 4B**), is being proposed here as a promising approach for improvements in on-target and in vitro ADME profile.

HDAC3 is a histone-modifying (deacetylation) enzyme belonging to class I HDAC family which acts as a crucial epigenetic regulator of inflammation in RA synovia, potentially through its activity on transcription factors (97). HDAC3 selective inhibitor blocked LPS-induced TLR signaling pathway resulting in reduced secretion of pro-inflammatory cytokines such as IL-6 and TNF-α by human monocytes and M1 macrophages (97). HDAC3 inhibition significantly suppressed IL-1β-induced inflammatory gene expression in RA FLS, at the same level of pan-HDAC inhibition, but without possible side effects such as thrombocytopenia (60). These strong evidences emphasize HDAC3’s potential as an important anti-cytokine target and its specific inhibition may ameliorate synovial inflammatory environment orchestrated by FLS and innate immune cells, characteristic of unresponsive or relapsed RA. A recent SAR study has reported a series of potent and selective HDAC3 inhibitors with 2-substituted benzamides as the zinc binding groups. While their selectivity is rationalized around the conformational flexibility of its catalytic site (PDB 4a69), inhibitor binding mode needs to be resolved to accelerate structure-based drug design efforts (98).

BET family proteins (BRD2, BRD3, BRD4, and BRDT) are epigenetic readers that recognize acetylated chromatin *via* their bromodomains and lead to transcriptional activation in responsive/targeted genes (99). An epidemiological study identified SNPs in BRD2 locus to be significantly associated with increased risk to RA (100). High expression of BRD2/3/4 in RA FLS and macrophages suggested their synergistic effect in the disease (101, 102). On studying their role in regulating synovial inflammation, their inhibition/knockdown blocked proliferation and TNF-α/IL-1β/TLR-induced production of MMPs and IL-6 in RA FLS (101). Another inhibitor study also displayed downregulation of TNF-α-induced NF-κB pathway *in vitro*, as well as efficacy in a CIA murine model (102). Furthermore, inhibitor JQ1 or BRD2/4 knockdown blocked VEGF-induced activation of p21-activated kinase 1 (PAK1) in endothelial cells, thereby suppressing angiogenesis (103). BET proteins are also implicated in diabetes, cardiovascular diseases and a variety of cancers, with 11 SMIs in ongoing clinical trials (104). With structures of binding modes such as PDB 5EK9 and 4MR4 available for both BRD2 and BRD4 respectively, *in silico* drug design using available SAR knowledge holds potential for developing BET inhibitors, specifically against BD2 domain to avoid toxicity associated with BD1 inhibition (105), and effectively prevent BET protein recruitment to inflammatory genes following cytokine stimulation (106). Interestingly, the possibility of dual kinase-bromodomain inhibition is being explored for developing cancer therapeutics (107–109). Computational SAR studies can be advanced in RA to tailor inhibitors for selectivity to BRD2/4 and disease-implicated kinase. Designing inhibitors with a polypharmacological profile might be a rational strategy to concurrently target both perturbed epigenetic landscape and dysregulated signaling underlying progression of chronic RA.

### 5.3 Leads From Metabolic Dysregulation

Activation of immune cells in RA disease requires changes in metabolic pathways owing to their increased demand for cellular expansion and biomass generation. Irrespective of whether it is the initial transition of naïve T-cells to effector cells; phenotypic transformation of FLS from a quiescent cell to an aggressive, metabolically active cell; activation of macrophages for overproduction of cytokines that promote immune cell infiltration; or the increasingly hypoxic (oxygen tension <1%) and nutrient‐deprived microenvironment that develops in the RA joint, metabolic alterations in RA differ from the response to acute stimulation in healthy individuals (74, 110). Also, metabolic signatures are cell type specific to match their immunological functions. Targeting these metabolic pathway perturbations to manipulate inappropriate immune responses in selective cell subsets is logical and offers an avenue for new therapeutics in RA as an alternative to immunosuppression. Important target leads in this group include Choline kinase alpha (CHKα) (111), Sphingosine kinase 1 (SPK1) (112) and hexokinase 2 (HK2) (113).

CHKα, which catalyzes phosphorylation of choline to phosphocholine, has a rate-limiting, regulatory role in phosphatidylcholine biosynthesis and amplification of cancer survival and invasion pathways of PI3K/AKT and MAPK (114, 115). CHKα is highly expressed in RA synovium intimal lining under pro-inflammatory conditions. Its inhibition by SMI (MN58b) suppressed cell migration and resistance to apoptosis of cultured fibroblast-like synoviocytes (FLS) by modulating PI3K-AKT and MAPK-ERK signaling pathways and abrogated joint inflammation and damage in a murine model of RA, thereby validating its candidature as a therapeutic target for RA (111). Furthermore, CHKα inhibition was shown to attenuate NLRP3 inflammasome activation and IL-1β production in stimulated macrophages (116). Apart from co-crystal structures of CHKα with ATP-competitive inhibitors, recent studies have led to the characterization of alternate proximal site (PDB 5W6O) on CHKα’s surface based on binding studies of its second-generation inhibitor TCD-717 (62) and elucidation of molecular interaction between CHKα’s non-catalytic site and SH3 domain of c-Src (117). These alternate allosteric sites open an alternate approach for new design of potent and specific CHKα inhibitors targeting the aggressive effector functions of RA FLS and macrophages in chronic RA.

Another potential target is sphingosine kinase 1 (SPK1), a cytosolic lipid kinase that regulates sphingolipid metabolic pathway. It phosphorylates sphingosine to make sphingosine-1-phosphate (S1P), a bioactive lipid involved in the pathogenesis of autoimmune diseases (112). The balance between the cellular levels of S1P and ceramide/sphingosine, so-called sphingolipid rheostat, acts as an important regulator of cell fate in other inflammatory diseases and cancers (118). *In vitro* and mice model studies have shown that SPK1 blockade or knockout suppressed synovial hyperplasia, leucocyte infiltration and joint erosion, by inhibiting downstream ERK or PI3K/AKT signaling in PBMC and FLS (119, 120). Apart from joint inflammation, plant extract-based small molecule, geniposide, also reduced the secretion of pro-angiogenic growth factors in AIA rats by reducing expression levels of VEGF, SPK1 and S1PR1 in FLS (121). Modulators of other members of SPK/S1P signaling pathway such as S1PR1 have shown toxicities including deleterious cardiovascular events, nasopharyngitis, urinary tract and viral infection and anemia among others (122, 123). The recently solved X-ray co-crystal structures of SPK1 such as PDB 3VZC and 4V24 (124, 125) and SAR studies of lipidic and non-lipidic small molecule inhibitors have established druggability of SPK1 in preclinical animal models of various diseases (126, 127). Off-target effects due to low specificity is however a concern. Identification of synergistic off-targets and development of compounds targeting the unique C4 domain of SPK1 would be an ideal strategy.

The increased bioenergetic demands of synovial fibroblasts and macrophages to transform into aggressive phenotypes are met by a shift from oxidative phosphorylation to glycolytic ATP production *via* HIF-1α (74). HK2, inducible isoform of hexokinase catalyzing the first step of glucose metabolism and a key glycolytic regulator downstream of HIF-1α, is specifically expressed in RA inflamed synovial lining as compared to osteoarthritis synovium (113). Inhibition or deletion of HK2 resulted in significant reduction in FLS activated and invasive phenotype *in vitro* and severity of bone and cartilage damage in murine arthritis model, thereby making it an attractive metabolic target in RA (113, 128). In addition to RASF, its inhibition led to suppression of inflammatory phenotype of synovial macrophages (129). Due to its limited expression in adult tissues than the ubiquitous form HK1, selective overexpression in RA inflamed synovium and its very small role in T cells, it can be selectively exploited for RA treatment without compromising global glucose metabolism and systemic immunosuppression (74). Co-crystal structures of inhibitor-bound C-terminal catalytic pocket of HK2 are available (PDB 5HG1, 5HFU). Although glucose binding sites are well conserved among the hexokinases, SAR analysis in a cancer-related study identified selective inhibitors of HK2 based on a hit molecule from high throughput screening (130). Preclinical inhibitors might serve as starting structures for developing potent HK2 inhibitor to target aggressive functions of RASF and macrophages.

### 5.4 Multi-Evidence Target Lead

**Figure 5:**
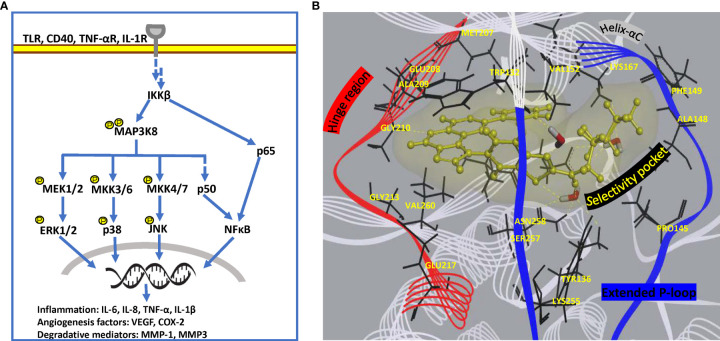
Pathway and structural details of MAP3K8 as potential drug target. **(A)** Diagrammatic representation of MAP3K8 signaling in innate and stromal cells for persistent inflammation. **(B)** Unique binding mode of a preclinical inhibitor in MAP3K8 kinase domain (PDB: 5IU2). Active site residues providing hydrogen bond and hydrophobic interactions are present in hinge region, helix-αC element as well as selectivity pocket formed due to extended flexible P-loop of kinase domain. Secondary structure of kinase domain is rendered as line ribbon including P-loop in blue, hinge region in red and rest of the domain in white. Key residues of the domain are rendered as black sticks, water molecules as sticks in element color and inhibitor as yellow ball and stick model. Soft surface of inhibitor is depicted in light yellow and hydrogen bond interactions as yellow dashed lines.

Search for strategically positioned kinase(s) regulating multiple clinically validated signalling cascades in a restricted cell-type manner is crucial for simultaneous targeting of multiple pathologies operational specifically in chronic rheumatoid joints. Challenges facing this approach include selectivity of inhibitors due to conservation in ATP-binding site across kinome and effect on additional roles of targeted signal transduction, if any. Drug development targeting a multi-evidence node, such as Mitogen-activated protein 3 kinase 8 (MAP3K8) seems promising for effective mitigation of disease. MAP3K8, also known as Tumor progression locus 2 (TPL2) or Cancer Osaka thyroid oncogene (COT), is a serine/threonine protein kinase that acts as a critical regulatory node in controlling inflammatory response *via* MAPK and NFκB cascades in a cell-type and stimulus-specific manner (131). Not only is it the exclusive regulator of ERK-mediated gene transcription downstream of proinflammatory signals, but it also activates synovial fibroblasts *via* JNK and NFĸB pathways (132). In addition, it modulates effector functions of macrophages and neutrophils through activation of PI3K/AKT and MKK3/MKK6-p38α axis, functional studies (131, 133). Treatment of dendritic cells with MAP3K8 inhibitors or siRNA provided evidence for MAP3K8 as the critical mediator of hypoxia-induced TNF-α secretion *via* p38/MAPK pathway (134). Since it regulates the expression of cytokines (TNF-α, IL-1β) which can reengage in original signaling cascade, it is a potent amplifier of proinflammatory signaling in multiple innate immune cell types (**Figure 5A**) (135). MAP3K8 inhibitors also thwart endothelial cell function in angiogenesis as well as block osteoclastogenesis (136). Animal studies have provided evidence of its involvement in Inflammatory bowel disease (IBD), psoriasis, multiple sclerosis, lupus, pancreatitis and lung inflammation (135). Notably, MAP3K8 rs1042058 is a significant IBD locus identified in transethnic GWAS (137). Therefore, multiple lines of evidence point at MAP3K8 inhibition as a powerful therapeutic strategy to dampen inflammatory perpetuation in a variety of disease settings. Most importantly, MAP3K8 has a unique kinase domain architecture wherein an extended and highly flexible P-loop covers the active site cleft due to a 15 amino acid insert (PDB 5IU2). This feature, not found in other human kinases, exposes a deeper flexible pocket which can be exploited for selective drug design (58), wherein traditional ATP-mimetic scaffolds may be extended for extra hydrophobic and hydrogen bonding interactions (**Figure 5B**). Although MAP3K8 is a relatively newer target for RA with few SAR inhibitors reported in preclinical studies (58, 138), a specific inhibitor, GS-4875, is advancing in clinical animal studies for multiple inflammatory diseases (139).

## 6 Summary and Perspectives

Current treatment convention in RA focuses on rapid and sustained suppression of the primary symptom of inflammation and at present there is no cure. DMARDs, biologics and small molecule therapeutics together have revolutionized RA treatment in the past two decades with tremendous improvement in clinical outcomes. Yet approximately 40-44% of RA patients fail to respond due to lack of efficacy or off-target effects demanding alternate treatment with drug combinations, but that has shown limited success. It is well known that synovium is the ultimate target tissue of inflammatory destruction in RA, basically resulting from a complex interplay between genetic and environmental factors. It is now emerging that molecular alterations in synovial biology lead to an imbalance of cellular homeostasis in turn leading to multiple pathologies. With such insights, a paradigm shift to synovial biology directed new therapeutics with permissible safety profiles is imminent. To achieve this, a deeper understanding of the spatiotemporal functional heterogeneity in predominant cell types such as fibroblasts and myeloid cells, responsible for sustained progression of local inflammatory and destructive environment in rheumatoid synovium, is a prerequisite. OMICS-based analysis of synovial cells in recent years have provided insights into genetic, epigenetic and metabolic alterations in activated synovial fibroblasts and macrophages, which get enriched in signaling pathways of migration, invasiveness and innate immunity. Genes strategically positioned in such signaling networks seem ideal as potential therapeutic targets without the risk of immunosuppression. Druggability of such proteins then emerges as the most important aspect which has been elaborated in this review.

In conventional drug development pipeline, availability of qualitative *in vitro* assays as well as knowledge from animal studies using gene knockouts, inactive knock-ins or inhibitors are pivotal criteria for the first step of target selection. Subsequent to this is the very elaborate, time consuming and expensive lead identification and optimization in preclinical and clinical studies. This process is expected to be expedited by application of computational methodologies to model novel drug design and test with experimental approaches. In this context, assessment of 3D structural parameters particularly for the development of SMI category with a favorable therapeutic compliance is essential. SMIs are a rather new option in autoimmune and inflammatory diseases compared to cancers and have multiple advantages of oral bioavailability; wide protection against pro-inflammatory cytokines; and cost-effectiveness over biologics. However, challenges in designing SMIs with target selectivity and minimal off-target effects cannot be undermined. Few notable SMIs including inhibitors against receptor-interacting serine/threonine protein kinase 1 (RIPK1) for psoriasis, MAP3K8/TPL2 for ulcerative colitis and TLR4 for autoimmune hepatitis are in the development pipeline (140).

In this review, promising targets for RA such as PADI4, SHP2, PI3Kδ, HDAC3, BRD2/4, CHKα, SPK1 and HK2 for possible inhibition by small molecules have been discussed. Notably, all of these are leads from contemporary GWASs, epigenomics and metabolic reprogramming in synovial fibroblasts and myeloid cells and deliberated on for their druggability (**Table 2**). In addition, targeted approach for alleviating dysregulated signaling networks *via* regulatory nodes (for example MAP3K8/TPL2) hold promise for inhibiting effector functions of multiple cell types and thereby suppressing parallel pathologies like angiogenesis, leukocyte trafficking, production of inflammatory mediators etc. Based on the selectivity pockets available for these promising leads, drug discovery can commence with computational methods such as structure-based pharmacophore modeling, virtual high throughput screening of chemical databases, scaffold hopping and ligand-based pharmacophore profiling (141).

**Table 2:** Properties of potential druggable targets in RA.

Leads from Multi-omics	Pathology	Cell Type Expression	Signaling pathway	Animal Model	3D Structures	Clinical Status
**MAP3K8/TPL2 (genomics, multiple evidences)**	Cell survival, production of inflammatory, degradative mediators, angiogenesis factors	Synovial fibroblasts, myeloid cells, neutrophils	MAPK pathways (ERK, p38 and JNK), NFĸB signaling	Tpl2 knockdown in CIA mice; inhibition in DSS-induced colitis mice model	5IU2 (catalytic site with adjacent selectivity pocket due to unique kinase domain structure)	SAR studies of preclinical molecules for RA; Clinical molecule for Ulcerative colitis
**PADI4 (genomics, transcriptomics, epigenomics)**	NET formation, inflammation, regulation of T-cell mediated immune response	Neutrophils, monocytes	Citrullination pathway	Inhibitor study in CIA murine model	4X8G, 4X8C (catalytic site)	Preclinical molecules for RA, SLE
**SHP2 (genomics and epigenomics)**	Survival and invasiveness of synovial lining FLS	FLS, myeloid cells	TNF-induced signaling through FAK and downstream MAPK (JNK, p38 and ERK) activation	Heterozygous *PTPN11* deletion study in K/BxN serum transfer arthritis in mice	5EHR (allosteric site with hydrophobic subpocket can be targeted for selectivity)	Preclinical non-competitive inhibitor for RA; Clinical molecules for solid tumors
**PI3Kδ (epigenomics)**	Synovial hyperplasia, cell mobility and activation	FLS in intimal lining, neutrophils, mast cells, B-cell, T-cell	AKT and RAC signaling	Inhibitor study in CIA rat model	4XE0 (Hydrophobic pocket adjacent to hinge region for selective lead design)	SAR studies in RA; FDA approved inhibitor for cancer; Phase-2 inhibitors for asthma, COPD
**HDAC3 (epigenomics)**	Inflammatory gene expression	FLS, M1 macrophages, monocytes	TLR signaling	Only *in vitro* studies	4A69 (apo-structure, conformationally flexible site)	SAR studies of preclinical molecules
**BRD2/4 (epigenomics, genomics)**	Transcriptional activation of MMP, IL-6,8; angiogenesis	FLS, macrophages, endothelial cells	TNF-α/IL-1β/TLR signaling, VEGF-PAK1 signaling	Inhibitor/siRNA study in CIA model	5EK9 (BRD2), 4MR4 (BRD4), BD2 domain selective; dual kinase/BRD inhibitors need to be explored	SAR studies of preclinical molecules for RA; 11 clinical trial molecules in other diseases
**CHKα (metabolic reprogram)**	Cell migration and proliferation, inflammation	FLS, macrophages	MAPK and PI3K/AKT signaling	Inhibitor study in K/BxN serum transfer arthritis in mice	5AFV, 4DA5 (ATP binding site), 5W6O (allosteric site)	Preclinical molecules for RA; Clinical molecules for solid tumors
**SPK1 (metabolic reprogram)**	Synovial hyperplasia, leucocyte infiltration inflammation	FLS	MAPK ERK, PI3K/AKT signaling	Knockdown in CIA, AIA	3VZC, 4V24 (catalytic site with selective features of C4 domain)	SAR studies of preclinical molecules for RA
**HK2 (metabolic reprogram)**	Cell activation, invasive phenotype	Synovial lining FLS	Inducible glucose metabolism	Deletion in K/BxN serum transfer arthritis model	5HG1, 5HFU (C-terminal catalytic pocket)	SAR studies of preclinical molecules for cancer

MAPK, mitogen-activated protein kinase; SAR, structure-activity relationship; PADI4, peptidyl arginine deiminase 4; SLE, systemic lupus erythematosus; FLS, fibroblast-like synoviocytes; TNF, tumor necrosis factor; SHP2, Src homology-2 domain-containing protein tyrosine phosphatase-2; PI3Kδ, phosphatidylinositide 3-kinase delta; HDAC3, histone deacetylase 3; BRD2/4, bromodomain and extra-terminal proteins; CHKa, choline kinase alpha; SPK1, sphingosine kinase 1; HK2, hexokinase 2; CIA, collagen-induced arthritis; AIA, antibody induced arthritis.

Combination therapy against the promising drug targets along with anti-cytokine biologics may be another optimistic approach to explore. This may be successfully realized by conducting single agent and combination trials involving clinically-relevant settings, large observational registries to evaluate effect-size and safety of intervention across distinct patient subgroups. Furthermore, restoring immune tolerance by development and transfer of autologous tolerogenic dendritic cells, regulatory T-cells and regulatory mesenchymal cells into rheumatoid joints is also being considered (142, 143). As some of the drug targets are common across other inflammatory or autoimmune diseases, repurposing of drugs/pharmacophores is also a feasible option. Finally, for effective RA management, efforts to identify biomarkers for early disease stratification as well as response prediction to old and new drugs seem inevitable.

## Author Contributions

GS and BKT contributed to conception and design of the review. GS wrote the first draft of the manuscript. BKT revised it critically for its content. GS and BKT contributed equally to manuscript revision, read, and approved the submitted version.

## Funding

This work was supported by Department of Biotechnology, Government of India, New Delhi through the "Centre of Excellence in Genome Sciences and Predictive Medicine" grant (#BT/COE/34/SP15246/2015-Phase-II) to BKT.

## Conflict of Interest

The authors declare that the research was conducted in the absence of any commercial or financial relationships that could be construed as a potential conflict of interest.

## Publisher’s Note

All claims expressed in this article are solely those of the authors and do not necessarily represent those of their affiliated organizations, or those of the publisher, the editors and the reviewers. Any product that may be evaluated in this article, or claim that may be made by its manufacturer, is not guaranteed or endorsed by the publisher.
